# Bezafibrate attenuates immobilization-induced muscle atrophy in mice

**DOI:** 10.1038/s41598-024-52689-x

**Published:** 2024-01-26

**Authors:** Satoshi Nakamura, Yuiko Sato, Tami Kobayashi, Akihito Oya, Astuhiro Fujie, Morio Matsumoto, Masaya Nakamura, Arihiko Kanaji, Takeshi Miyamoto

**Affiliations:** 1 Department of Orthopedic Surgery, Keio University School of Medicine, 35 Shinano-Machi, Shinjuku-Ku, Tokyo, 160-8582 Japan; 2 Department of Orthopedic Surgery, Kumamoto University, 1-1-1 Honjo, Chuo-Ku, Kumamoto, 860-8556 Japan

**Keywords:** Phenotypic screening, Experimental models of disease

## Abstract

Muscle atrophy due to fragility fractures or frailty worsens not only activity of daily living and healthy life expectancy, but decreases life expectancy. Although several therapeutic agents for muscle atrophy have been investigated, none is yet in clinical use. Here we report that bezafibrate, a drug used to treat hyperlipidemia, can reduce immobilization-induced muscle atrophy in mice. Specifically, we used a drug repositioning approach to screen 144 drugs already utilized clinically for their ability to inhibit serum starvation-induced elevation of Atrogin-1, a factor related to muscle atrophy, in myotubes in vitro. Two candidates were selected, and here we demonstrate that one of them, bezafibrate, significantly reduced muscle atrophy in an in vivo model of muscle atrophy induced by leg immobilization. In gastrocnemius muscle, immobilization reduced muscle weight by an average of ~ 17.2%, and bezafibrate treatment prevented ~ 40.5% of that atrophy. In vitro*,* bezafibrate significantly inhibited expression of the inflammatory cytokine *Tnfa* in lipopolysaccharide-stimulated RAW264.7 cells, a murine macrophage line. Finally, we show that expression of *Tnfa* and *IL-1b* is induced in gastrocnemius muscle in the leg immobilization model, an activity significantly antagonized by bezafibrate administration in vivo. We conclude that bezafibrate could serve as a therapeutic agent for immobilization-induced muscle atrophy.

## Introduction

Fragility fractures, sarcopenia, frailty, and muscle atrophy are conditions that reduce activity of daily living (ADL) and increase risk of the elderly becoming bed-ridden. Disuse and immobilization cause osteoporosis and muscle atrophy, which not only worsen ADL, quality of life, and healthy life expectancy, but also impair life expectancy. Muscle atrophy is also a risk factor for falls, which can lead to further disuse from fragility fractures and a negative cycle of disuse.

Muscle atrophy is mediated by myostatin^1^, ubiquitin/proteasome^2^, calpain^3^ and autophagy-lysosome^4^ pathways. Smad2/3-induced Atrogin-1 and MuRF1 in the myostatin pathway are known to suppress muscle atrophy by knockout^5^. We previously reported increased levels of Smad2/3 protein in muscle tissue in a model of immobilization-induced muscle atrophy in mice^6^ and that knockout of Schwann cell vitamin D receptor in this mouse model exacerbated muscle atrophy by increasing *Tnfa* and *Il-1b* expression^7^. Calpain pathways also function in muscle atrophy resulting from disuse; denervation reportedly increases mitochondrial Ca^2+^ levels in skeletal muscle^8^ and calcium channel blockers reduce immobility-related muscle atrophy^9^. Previous analyses of tail-suspension and denervation models indicated that neuronal nitric oxide synthase (nNOS) located in the sarcolemma moves into the cytoplasm to produce NO, in turn regulating Foxo3a-dependent induction of *Atrogin-1* and *MuRF1* expression and subsequent muscle atrophy^10^. On the other hand, muscle protein synthesis is regulated by IGF-1/AKT/mTOR signaling, and muscle atrophy due to unloading is induced by Cbl-b downregulation of IGF-1 signaling^11^. NO has also been shown to inhibit IGF-1/AKT/mTOR signaling^12^.

Several therapeutic agents reportedly antagonize muscle atrophy, including growth hormone, Insulin like Growth Factor-1 (IGF-1), androgens, Selective Androgen Receptor Modulators (SARMs)^13–15^, SGLT2 inhibitors^16^, antimyostatin antibodies^17,18^, beta stimulants^19^, and vitamin D^20^. In cases of IGF-1 deficiency, replacement therapy is available clinically, but expensive. In human subjects, treatment with SARMs reportedly increases muscle weight but not muscle strength or physical function^14^ and apparently does not promote prostate enlargement or seminal vesicle enlargement, as is seen in similarly treated male rats and mice^15^. The anti-myostatin antibody domagrozumab reportedly increases muscle weight in mice but does not promote significant changes in muscle cross-sectional area^17^, and the activin receptor antagonist bimagrumab increases muscle mass and grip strength but only partially increases walking speed and 6-min walking distance^18^. However, analysis of a mouse model of global myostatin deficiency in adults indicates immobilization-induced muscle atrophy comparable to that seen in wild-type mice^6^. Thus overall, there is currently no consensus on drug therapy for muscle atrophy.

Drug repositioning is a means to identify drugs with therapeutic effects in diseases different from those the drug was clinically approved for. Drugs identified through this method have advantages in that they have already been evaluated for side effects and safety, and their production and sales channels are established, so hurdles to commercialization are low and drug costs are often lower^21^. Examples of past successes include the repurposing of aspirin from an analgesic to an antiplatelet drug^22^, of thalidomide from a sleeping pill to treatment for multiple myeloma^23^, and of raloxifene from breast cancer treatment to use against osteoporosis^24^.

Here, using a similar strategy, we screened a library of 144 drugs already in clinical use for their ability to inhibit immobilization-induced muscle atrophy in mice. We identified several promising candidates, among them bezafibrate, a drug used to treat hyperlipidemia, which we show prevents immobilization-induced muscle atrophy in mice, both in vitro and in vivo.

## Materials and methods

### Drug library

A drug library containing 130 oral and 14 topical prescription medications was provided as previously described^25^. Dimethyl sulfoxide served as the solvent.

### Cell culture

#### C2C12 cells

C2C12 cells were purchased (CRL-1772, ATCC, Manassas, VA), maintained in growth medium (DMEM supplemented with 10% FCS, penicillin (50 units/ml), and streptomycin (100 µg/ml)) and maintained at 37 °C in a humidified 5% CO_2_ atmosphere. C2C12 cells were cultured 24 h in DMEM with 10% FBS, or without FBS in serum starvation conditions, in the presence or absence of candidate drugs (1 µM). C2C12s were also cultured 24 h in DMEM without FBS and stimulated with drug No. 001 (1 µM or 0.05 µM) or No. 003 (1 µM or 10 µM) for the last 6 h. Cmax in humans is 0.05 µM and 10 µM for drugs No. 001 and 003, respectively.

#### Myotubes

C2C12 myoblasts were cultured 72 h in DMEM with 2% horse serum, penicillin and streptomycin to induce myotube differentiation. Myotubes were cultured in DMEM with or without horse serum as serum starvation for 24 h and stimulated with 1 µM of candidate drugs or 10 or 50 ng/ml of recombinant human Insulin-Like Growth Factor-1 (rhIGF-1; R&D SYSTEMS, 291-G1, Minneapolis, MN) for the last 6 h.

#### RAW 264.7 cell

RAW 264.7 cells were purchased (TIB-71, ATCC) and cultured as described^26^. Briefly, cells were cultured 24 h in DMEM containing 10% FBS, 1% GlutaMAX, penicillin and streptomycin with or without 10 ng/ml LPS (Sigma-Aldrich, St. Louis, MO, USA) and stimulated 24 h with 2, 10, 50 or 250 µM bezafibrate.

### Quantitative RT-PCR

Total RNAs were isolated from cultured cells using the RNeasy mini kit (Qiagen, Hamburg, Germany), and cDNA was synthesized using oligo (dT) primers. Realtime PCR was performed using SYBR Premix ExTaq II reagent and a DICE Thermal cycler (Takara Bio Inc.) as described^27^. *Gapdh* expression served as an internal control.

Primer sequences were:

*Gapdh* forward: 5′-ACCCAGAAGACTGTGGATGG-3′.

*Gapdh* reverse: 5′-TTCAGCTCTGGGATGACCTT-3′.

*Atrogin-1* forward: 5′-GAGACCATTCTACACTGGCAGCA-3′.

*Atrogin-1* reverse: 5′-GTCACTCAGCCTCTGCATGATGT-3′.

*MuRF1* forward: 5′-ACCTGCTGGTGGAAAACATCATT-3′.

*MuRF1* reverse: 5′-AGGAGCAAGTAGGCACCTCACAC-3′.

*Smad2* forward: 5′- CAGGACGGTTAGATGAGCTTGAGA-3′.

*Smad2* reverse: 5′- CCCACTGATCTACCGTATTTGCTG-3′.

*Smad3* forward: 5′- GAAACCAGTGACCACCAGATGAAC-3′.

*Smad3* reverse: 5′- CGTAGTAGGAGATGGAGCACCAGA-3′.

*Tnfa* forward: 5′-CTTCTGTCTACTGAACTTCGGG-3′.

*Tnfa* reverse: 5′-CAGGCTTGTCACTCGAAT TTTG-3′.

*Il-1β* forward: 5′-AAGTTGACGGACCCCAAA AGAT-3′.

*Il-1β* reverse: 5′-AGCTCTTGTTGATGTGCTGCTG-3′.

*Il-6* forward: 5′-GTCCTTAGCCACTCCTTCTG-3′.

*Il-6* reverse: 5′-CAAAGCCAGAGTCCTTCAGAG-3′.

### Mice

C57BL/6 female mice were purchased from Sankyo Laboratory (Tokyo, Japan). Mice were housed up to five per cage and maintained on a 12-h light–dark cycle. Mice were maintained under specific pathogen-free (SPF) conditions in animal facilities certified by the Keio University Institutional Animal Care and Use Committee. All animal experiments were carried out in accordance with the Institutional Guidelines on Animal Experimentation at Keio University, and animal experiment protocols were approved by the Keio University Institutional Animal Care and Use Committee.

### In vivo effects of candidate drugs

Mice were injected with No. 001, No 003 (bezafibrate; Wako, 022-16091, Osaka, Japan) or vehicle (ethanol) intraperitoneally for eight days daily starting at nine-weeks of age. Each group contained five mice, and animals were treated daily with 4 µg of No.001, 160 µg of No.003 or vehicle (ethanol) administered intraperitoneally. Administration of 4 µg (No.001) or 160 µg (No.003) to a 20 g mouse corresponds to administration of 10 mg (No.001) or 400 mg (No.003) to a 50 kg human. Ethanol served as the solvent for bezafibrate in vivo. 8 µl of bezafibrate stock solution solved in ethanol or ethanol as vehicle were administered with 100 µl of PBS. Left hind limbs of mice were either immobilized or denervated on day two (of drug treatment), and mice were sacrificed on day nine. To immobilize hind limbs, midfoot parts of the left hind limbs were fixed at the maximum flexion position of hip, knee and ankle joints using AUTOCLIP 9 mm and CLIP Applier. Right hind limbs, which were not immobilized, served as controls. For denervation, left sciatic nerves were cut, and a 1-mm portion of nerve was removed to denervate the gastrocnemius muscle. Sham (control) surgery involved a skin incision into the right hind limb. For bezafibrate administration, the same fixation and denervation procedures were performed twice, and a total of 50 mice was used. This study is reported in accordance with ARRIVE guidelines.

### Histology

4-μm paraffin cross sections of gastrocnemius and quadriceps muscles were stained with hematoxylin and eosin. We analyzed fiber cross-sectional area (CSA) at three randomly selected locations with BioRevo (Keyence, Osaka, Japan), as others have shown that fiber counts from 3–4 fields provided a reasonable prediction of total fiber number^28^. In the vehicle group the number of myofibers evaluated per mouse was 178–294 or 132–401 in gastrocnemius or quadriceps muscle, respectively; in the bezafibrate group they were 164–266 or 134–230 in gastrocnemius or quadriceps muscle, respectively.

### Immunohistochemistry

Gastrocnemius muscles were embedded in paraffin and cut into 4 μm sections. After blocking 1 h with 5% bovine serum albumin/PBS containing 0.1% Tween 20 at room temperature, sections were incubated with Alexa Fluor 488 anti-mouse F4/80 antibody (1:100 dilution; 123120, BioLegend, San Diego, CA) and anti-TNF alpha antibody (1:100; ab6671, Abcam, Cambridge, UK) overnight at 4 °C. After three PBS washes, sections were stained 1 h with F(ab')2-Goat anti-Rabbit IgG (H + L) Cross-Adsorbed Secondary Antibody, Alexa Fluor 546 (1:200; Invitrogen, A-11071, Waltham, MA) at room temperature. DAPI (1:5000; Wako Pure Chemicals Industries, Osaka, Japan) was used for a nuclear stain. The number of F4/80/TNFα double-positive cells and DAPI positive nuclei was determined in five randomly selected 500-μm squares using a fluorescence microscope (BioRevo, Keyence, Osaka, Japan).

### Western blotting

Lysates were obtained from frozen minced gastrocnemius muscle using RIPA buffer (1% Tween 20, 0.1% SDS, 150 nM NaCl, 10 mM Tris–HCl (pH 7.4), 1 mM phenylmethylsulfonyl fluoride (#P7626, Sigma-Aldrich Co. LLC, St. Louis, MO), 50 μg/ml aprotinin (#A1153, Sigma-Aldrich Co. LLC), 100 μg/ml leupeptin (#L2884, Sigma-Aldrich Co. LLC), 1 mM Na3VO4 (198–09752, FUJIFILM Wako Pure Chemical Corporation, Osaka, Japan), and 25 μM pepstatin A (#P5318, Sigma-Aldrich Co. LLC)). 30 μg protein was loaded onto and run on 12.5% SDS–PAGE gels (e-PAGEL, ATTO Corporation, Tokyo, Japan) and then transferred to PolyVinylidene DiFluoride (PVDF) membranes (Immobilon, Merck KGaA, Darmstadt, Germany). Membranes were blocked with buffer containing 10 mM Tris·HCl (pH 7.4), 150 mM NaCl, 0.1% Tween 20, and 5% skim milk or bovine serum albumin and then incubated overnight with each primary antibody at 4 °C. Membranes were then incubated with HRP-conjugated Goat anti-Rabbit IgG (1:5,000; G21234, Thermo Fisher Scientific, Waltham, MA) as secondary antibody, and immune complexes were visualized using the ECL Western Blotting Analysis System (RPN2235, GE Healthcare, Chicago, IL).

Primary antibodies used to detect proteins were anti-phospho-Smad2 (1:1,000; #3101, Cell Signaling Technology, Inc., Beverly, MA), anti-phospho-Smad3 (1:1,000; #9520, Cell Signaling), anti-Smad2/3 (1:1,000; #3102, Cell Signaling) and anti-Gapdh (1:20,000; GTX100118, GeneTex, Irvine, CA). ImageJ was used for quantification, as described^29^.

### Statistical analysis

Data are shown as means ± SD. Statistical significance was evaluated by Student’s t-test or one-way ANOVA and a Tukey post hoc test. A probability of less than 5% was considered statistically significant (*P < 0.05; **P < 0.01; ***P < 0.001; ns, not significant).

## Results

### Drug screening and selection of candidate drugs that block muscle atrophy

**Figure 1:**
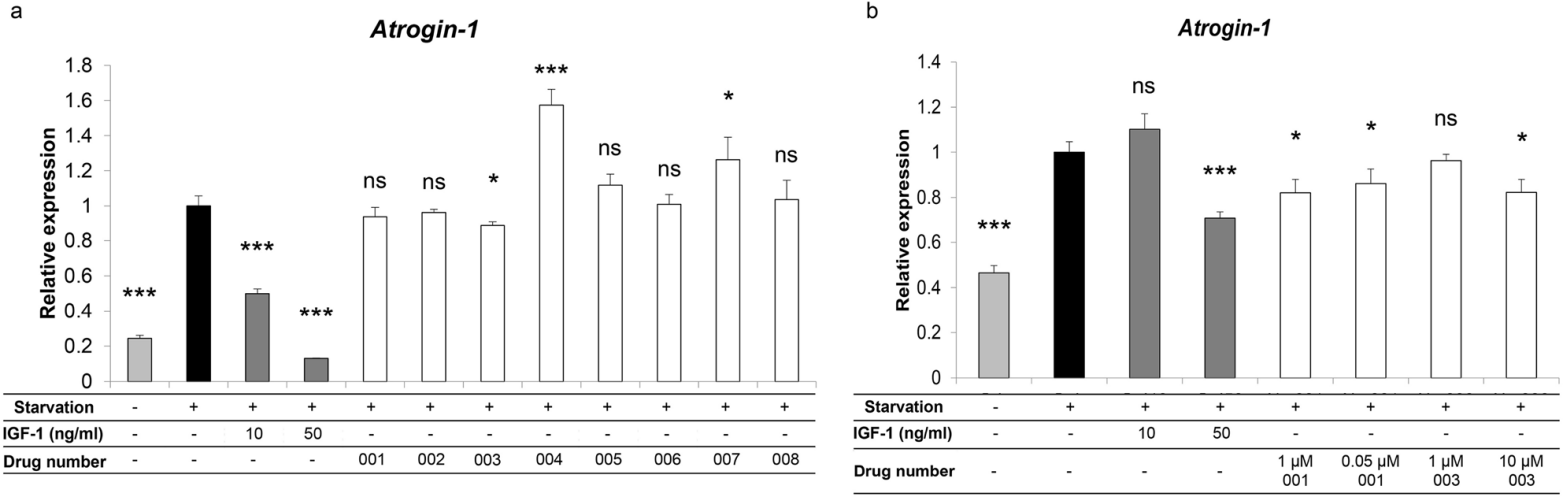
Effects of existing drugs on serum starvation-induced *Atrogin-1* expression in C2C12 myoblasts. (**a**) C2C12 cells were cultured for 72 h in DMEM with 2% horse serum to induce myotube formation. Subsequently, myotubes were cultured with or without (serum starvation) 2% horse serum for 24 h and stimulated with indicated drugs (each 1 µM) or 10 or 50 ng/ml rhIGF-1 for the last 6 h. *Atrogin-1* expression was analyzed by realtime PCR. Data represent mean *Atrogin-1* expression relative to *Gapdh* ± SD. Serum starvation without drugs or IGF-1 served as controls (each n = 3, *P < 0.05, ***P < 0.001 by Student’s t-test). (**b**) C2C12 cells were cultured 24 h in DMEM with or without (serum starvation) 10% FBS in the presence or absence of indicated concentrations of drugs No.001 or 003 for the last 6 h. *Atrogin-1* expression was then analyzed by realtime PCR. Data represent mean *Atrogin-1* expression relative to *Gapdh* ± SD. Serum starvation without drugs served as controls (each n = 3, *P < 0.05, ***P < 0.001 by Student’s t-test).

We initially tested 144 clinically available drugs for ability to inhibit expression of the muscle catabolic gene *Atrogin-1* using the myoblast C2C12 cell line. Serum starvation of this line stimulates *Atrogin-1* expression and represents an in vitro pseudo-muscle atrophy model^30^. For our analysis we added each drug at a concentration of 1 µM to C2C12 cell cultures for 24 h (Fig. S1) and identified eight drugs that suppressed *Atrogin-1* expression, but none significantly. Nonetheless, the lowest expression was seen in the presence of drug No. 001. We then differentiated C2C12 cells into myotubes by culturing them 72 h in medium containing 2% horse serum^31^, and followed that with 24 additional hours of serum starvation and 6 h of stimulation with each of eight candidate drugs (Fig. 1a). For the second screen, we used 1 μM concentrations of candidate drugs to assess effects on myotubes (Fig. 1a). As a positive control, we used 10 or 50 ng/ml of recombinant human IGF-1 (rhIGF-1), which is known to reduce *Atrogin-1* expression in C2C12 cells^6^. *Atrogin-1* expression following serum starvation of C2C12 myotubes was significantly inhibited by 12% by drug No. 003 (Fig. 1a), the PPARα/γ/δ pan-agonist bezafibrate. Therefore, we subjected drugs No. 001 and No. 003 to further screening. To do so, we serum-starved C2C12 cells 24 h without myotube induction and then added each drug to cultures for 6 h at human Cmax concentrations (0.05 µM for No. 001 and 10 µM for No. 003) as well as 1 µM. Both No. 001 and No. 003 significantly inhibited *Atrogin-1* expression in C2C12 cells at Cmax concentrations (Fig. 1b).

### Bezafibrate significantly inhibits immobilization-induced muscle atrophy in mice

**Figure 2:**
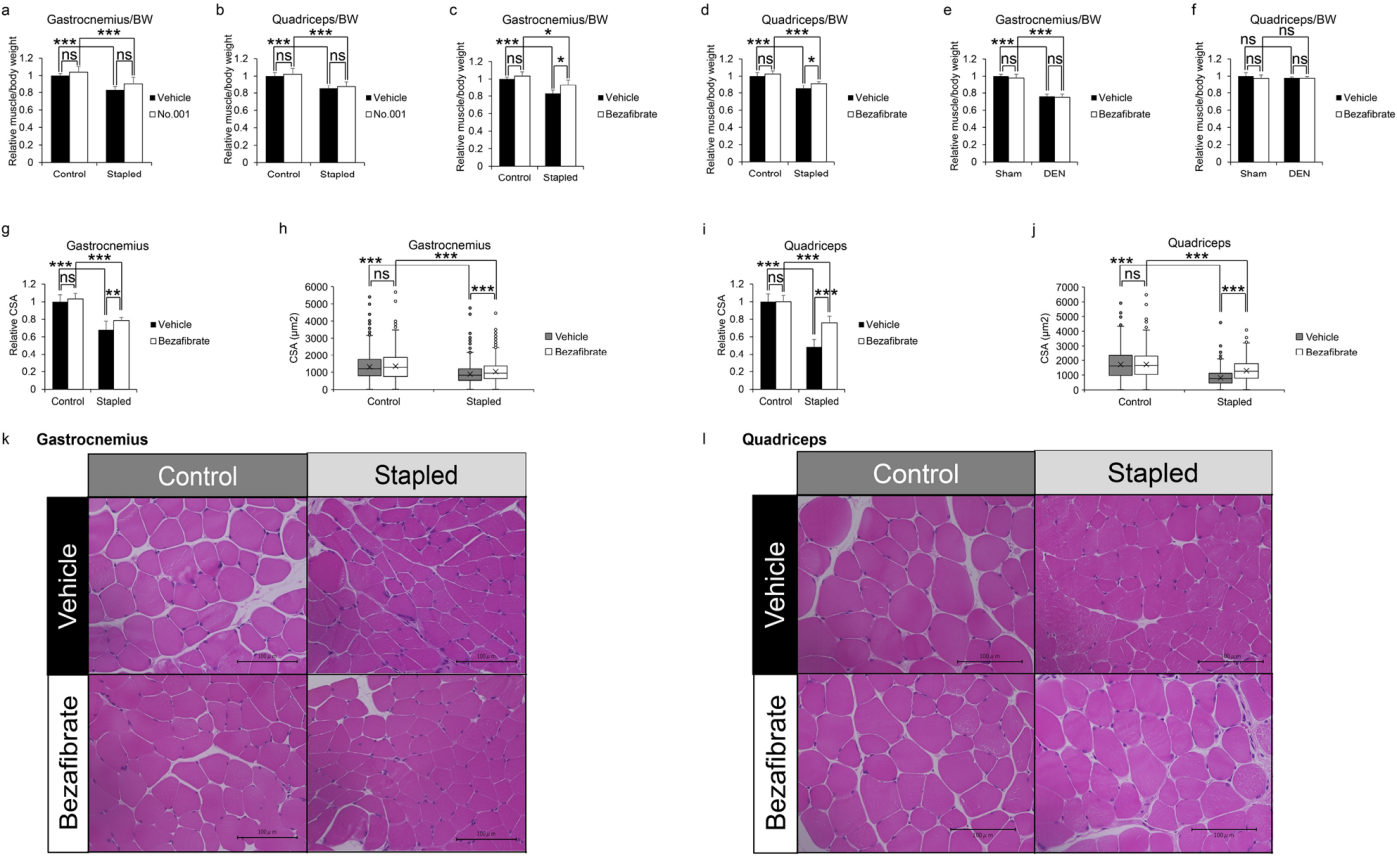
Bezafibrate reduces immobilization- but not denervation-induced muscle atrophy in mice. Nine-week-old C57BL/6 mice were treated with No.001, No.003 (bezafibrate) or vehicle once daily for eight days, and staple fixation or sciatic nerve denervation surgery (DEN) was performed on left hind limbs on day two. On day nine, mice were sacrificed and gastrocnemius and quadriceps muscles were harvested. Right hind limbs served as controls (sham-operated) (n = 5, each group). Wet weights of gastrocnemius (**a**,**c**) and quadriceps (**b**,**d**) muscle adjusted to body weight in the stapled plus drug conditions (No.001, (**a**,**b**); No.003, (**c**,**d**)) versus the control side were analyzed. Wet weights of gastrocnemius (**e**) and quadriceps (**f**) muscle adjusted to body weight in the denervated plus No.003 condition versus the sham side are shown. Representative data of two independent experiments are shown. (*P < 0.05; ***P < 0.001 by Student’s t-test). (**g**–**j**) Relative mean cross-sectional areas (CSA) of gastrocnemius (**g**) and quadriceps (**i**) muscles from mice treated with bezafibrate, with or without staple fixation. (**P < 0.01; ***P < 0.001 by Student’s t-test). Boxplot of CSA of gastrocnemius (**h**) and quadriceps (**j**) muscles from same mice. CSA was measured at three randomly selected regions. The number of myofibers evaluated per mouse in the vehicle group was 178–294 or 132–401 in gastrocnemius or quadriceps, respectively. In the bezafibrate group it was 164–266 or 134–230 in gastrocnemius or quadriceps. (**g**–**j**). (**k**,**l**) Hematoxylin and eosin staining of gastrocnemius (**k**) and quadriceps (**l**) muscles. Scale bar, 100 µm.

For in vivo analysis, we divided nine-week-old C57BL/6 female mice into three treatment groups—with No. 001, No. 003 (bezafibrate) or Vehicle (ethanol)—to create an immobilization-induced muscle atrophy model by stapling of lower extremities (stapled) or denervation of the sciatic nerve (DEN), and administered candidate drugs or ethanol/vehicle intraperitoneally once daily for eight days, starting one day before immobilization. The daily dose for a 50 kg human was converted to a mouse equivalent (for a 20 g mouse) of 4 μg for No.001 and 160 μg for bezafibrate (Fig. 2). After eight days of treatment, muscle weight of both gastrocnemius (Fig. 2a and c) and quadriceps (Fig. 2b and d) was significantly reduced in the vehicle group, but that reduction was significantly inhibited in the bezafibrate group, an effect not seen after administration of drug No.001. The average loss in muscle weight due to immobilization was ~ 17.2% in gastrocnemius muscle. However, bezafibrate treatment blocked ~ 40.5% of that loss (Fig. 2c). Similarly, quadriceps muscle also showed an average loss of ~ 14.8% of muscle weight following immobilization, but bezafibrate treatment prevented ~ 26.1% of this loss (Fig. 2d). In parallel analyses, bezafibrate treatment did not block gastrocnemius muscle weight loss following denervation (Fig. 2e). Moreover, denervation did not alter weight of quadriceps muscle, which is not a sciatic nerve target (Fig. 2f). Histological evaluation revealed that, relative to vehicle controls, bezafibrate treatment increased myofiber cross-sectional area in the gastrocnemius (Fig. 2g, h and k) and quadriceps (Fig. 2i, j and l) muscles on the immobilized side. We measured the minimum Feret diameter to exclude the possibility that muscle cross sections were obliquely cut (Fig. S2). In muscle cross sections we also observed a significant increase in the minimum Feret diameter of stapled gastrocnemius (Fig. S2a and b) or quadriceps (Fig. S2c and d) in the bezafibrate group.

### Bezafibrate treatment decreases expression of inflammatory cytokines in immobilized gastrocnemius muscle

**Figure 3:**
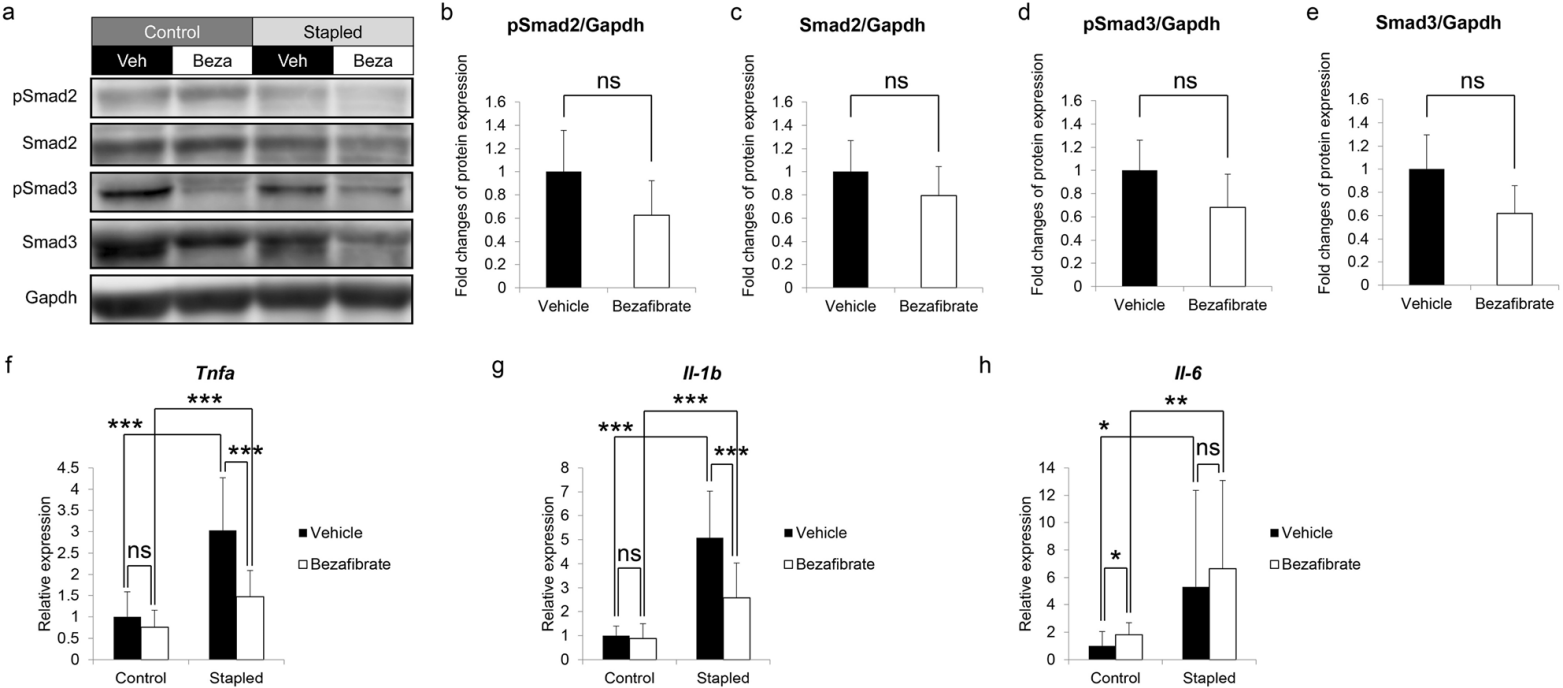
Bezafibrate treatment decreases *Tnfa* and *Il-1b* expression but does not inhibit Smad2/3 protein accumulation or phosphorylation in stapled gastrocnemius muscles. (**a**–**e**) Nine-week-old C57BL/6 mice were treated with bezafibrate or vehicle once daily for eight days. Staple fixation was performed on left hind limbs on day two of drug treatment. On day nine, mice were sacrificed and gastrocnemius muscles were harvested. Accumulation of Smad2, phosphorylated Smad2 (pSmad2), Smad3 and phosphorylated Smad3 (pSmad3) proteins in same muscles, as detected by western blotting (**a**). Representative images are shown. Accumulation of pSmad2 (**b**), Smad2 (**c**), pSmad3 (**d**), and Smad3 (**e**) protein on the fixed side of gastrocnemius muscle based on ratios of indicated proteins to Gapdh protein, based on ImageJ. Data represent mean ± SD, and vehicle groups served as controls (each group n = 3. ns; not significant by Student’s t-test). Relative expression of *Tnfa* (**f**), *Il-1b* (**g**) and *Il-6* (**h**) in the same muscles, based on realtime PCR (**f**–**h**). Data represent mean gene expression relative to *Gapdh* ± SD (each n = 5, *P < 0.05; **P < 0.01; ***P < 0.001 by Student’s t-test).

We next created the immobilization-induced muscle atrophy model in nine-week-old C57BL/6 female mice and administered bezafibrate or vehicle (ethanol) for eight days, starting a day before immobilization (Fig. 3). Based on western blotting, relative to vehicle-treated controls, levels of phosphorylation and accumulation of Smad2/3 protein in gastrocnemius muscle on the fixed side tended to decrease following bezafibrate treatment, although those differences were not statistically significant (Fig. 3b–e). Similarly, expression levels of the muscle catabolic genes *Atrogin-1* (Fig. S3a), *MuRF1* (Fig. S3b) and *Smad2* (Fig. S3c) in gastrocnemius muscle were unchanged by bezafibrate treatment, although bezafibrate treatment significantly reduced *Smad3* transcript levels in gastrocnemius muscle (Fig. S3d) in staple fixation-induced muscle atrophy models. Furthermore, expression of the inflammatory cytokines *Tnfa* (Fig. 3f), *Il-1b* (Fig. 3g) or *Il-6* (Fig. 3h) significantly increased in gastrocnemius muscle on the immobilized side (Stapled) of vehicle-treated mice, while comparable bezafibrate-treated mice showed significant inhibition of *Tnfa* and *Il-1b* but not *Il-6* expression.

### Bezafibrate inhibits LPS*-*dependent *Tnfa* expression in RAW cells and in vivo TNFα protein expression in gastrocnemius muscle macrophages following immobilization

**Figure 4:**
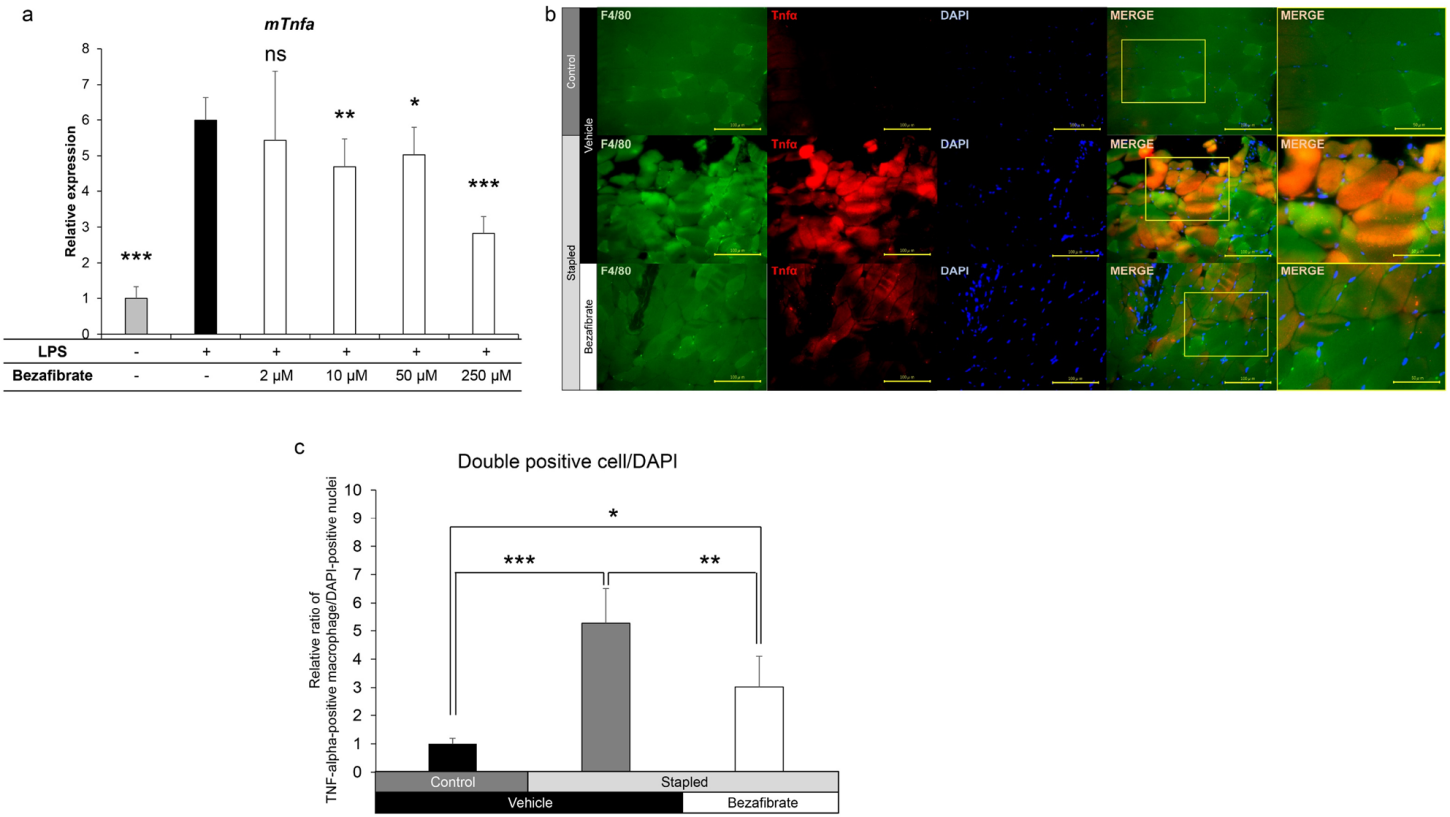
LPS-induced Tnfa expression in macrophages decreases after bezafibrate treatment. (**a**) RAW 264.7 cells were cultured 24 h with or without 10 ng/ml LPS in the presence or absence of indicated concentrations of bezafibrate. Relative *Tnfa* expression was then analyzed by realtime PCR. Data represent mean *Tnfa* expression relative to *Gapdh* ± SD (each n = 3, *P < 0.05; **P < 0.01; ***P < 0.001 by Student’s t-test). (**b**) Nine-week-old C57BL/6 mice were treated with bezafibrate or vehicle once daily for eight days. Staple fixation was performed on left hind limbs on day two of drug treatment. On day nine, mice were sacrificed and gastrocnemius muscles were harvested. Specimens were stained with Alexa Fluor 488-conjugated rat anti-mouse F4/80 and rabbit anti-mouse TNFα, followed by Alexa546-conjugated goat anti-Rabbit IgG. Nuclei were stained with DAPI. Sections were observed under a fluorescence microscope and all magnifications are 400x. Scale bar, 100 µm. In merged samples, images shown in the rightmost column are higher magnifications of squared areas in the adjacent image. In the latter, scale bars indicate 50 µm. (**c**) Shown is the ratio of the number of F4/80/TNFα double-positive cells and the number of DAPI-positive nuclei measured at five randomly selected locations. Data represent mean ratios relative to numbers observed in the vehicle (control) group ± SD (n = 5, *P < 0.05; **P < 0.01; ***P < 0.001 by one-way ANOVA and Tukey post hoc test).

Next, in order to analyze the effects of bezafibrate on the inhibition of *Tnfa* expression, we utilized RAW 264.7 cells, macrophage-like cells, and cultured them with LPS to induce *Tnfa* expression in the presence or absence of bezafibrate for 24 h (Fig. 4). As anticipated, *Tnfa* expression in vitro was significantly upregulated after 24 h of LPS stimulation in RAW 264.7 cells not treated with drug; however, after 24 h of bezafibrate treatment, we observed significant and dose-dependent inhibition of LPS-dependent *Tnfa* expression (Fig. 4a).

To assess macrophage accumulation, we analyzed the gastrocnemius muscles from bezafibrate and vehicle group (Fig. 4b). We observed accumulation of F4/80-positive macrophages and expression of TNFα protein in immobilization-induced atrophied gastrocnemius muscle of vehicle-treated mice (Fig. 4b). Significantly, TNFα expression in macrophages from atrophied gastrocnemius muscle was inhibited in mice that had been administered bezafibrate (Fig. 4b). The ratio of the number of F4/80/TNFα double-positive cells to the number of DAPI-positive nuclei was significantly increased by immobilization and significantly decreased by bezafibrate administration (Fig. 4c).

## Discussion

Muscle homeostasis is regulated by a delicate balance between muscle synthesis promoted by insulin like growth factor-1 (IGF-1)^32^ and muscle catabolism. We previously reported a mouse model of sarcopenia supporting the idea that reducing serum IGF-1 levels in adult mice causes muscle atrophy^27^. Muscle catabolism is mediated by various factors including muscle disuse, limb immobilization or steroid use^33^. It was previously shown that immobilization-induced muscle atrophy is promoted by nuclear translocation of a phosphorylated Smad2/3/4 trimer, which induces expression of the muscle catabolic genes *Atrogin-1* and *MuRF1*^1^, and also by the ubiquitin proteasome pathway^2^. We previously reported increases in Smad2/3 protein levels in atrophied muscle following limb immobilization and showed that muscle-specific Smad2/3 deficient mice are resistance to immobilization-induced muscle atrophy^6^. We also demonstrated that vitamin D receptor knockout in Schwann cells induces *Tnfa* and *Il-1b* expression in immobilized muscle and exacerbates muscle atrophy^7^. In the present study, we show that treatment with the drug bezafibrate downregulates *Atrogin-1* expression in cultured myoblasts and antagonizes immobilization-induced muscle atrophy in mice. We found that bezafibrate treatment did not significantly alter Smad2/3 phosphorylation and protein levels or *Atrogin-1* mRNA expression in immobilized gastrocnemius muscle. However, bezafibrate treatment decreased TNFα expression by LPS-stimulated macrophages in vitro as well as TNFα expression in muscle immobilized in vivo.

The PPARα/γ/δ pan-agonist bezafibrate is used as a therapeutic agent to treat hypercholesterolemia^34^. Bezafibrate also reportedly inhibits osteosarcoma cell growth when combined with medroxyprogesterone^35^ and improves motor performance in Parkinson's disease^36^. Bezafibrate has also been shown to have anti-inflammatory effects on retinal microvessels^37^ and in Covid-19 patients^38^. In the present study, *Tnfa* and *Il-1b* expression was reduced by bezafibrate, but not that of *Il-6*. In our previous report of a neural crest cell-specific vitamin D receptor knockout, we also observed decreases in *Tnfa* and *Il-1b* but not *Il-6*^7^. Although these differences remain unclear, we note that IL-6 is a myokine and an inflammatory cytokine secreted by muscle contraction^39^.

Bezafibrate has been approved to treat human diseases in England, Canada and Japan, and some muscle damage is reported in human subjects during chronic treatment^40^. Non-specific gastrointestinal disorders, elevated liver enzymes and rhabdomyolysis have also been reported as side-effects of fibrates, including bezafibrate^41^.

One review reports that high triglycerides and low HDL cholesterol are sarcopenia risk factors^42^. Some lipid mediators (such as polyunsaturated fatty acids, α-linolenic acid, eicosapentaenoic acid, docosapentaenoic acid and docosahexaenoic acid) have anti-inflammatory effects and thus could be useful to treat sarcopenia^43^. Furthermore, in the elderly, dietary omega-3 fatty acid supplementation increases mTOR and p70s6k phosphorylation in muscle and promotes increases in muscle protein synthesis^44^. We observed that creatine kinase, triglyceride and LDL-cholesterol levels were reduced in bezafibrate relative to vehicle-treated mice (Fig S4), but those differences were not significant.

Although bezafibrate treatment reduced *Atrogin-1* expression in C2C12 myoblasts and myotubes, we currently do not know why bezafibrate did not inhibit *Atrogin-1* expression in immobilized gastrocnemius muscle, nor is it clear why bezafibrate inhibited immobilization-induced muscle atrophy conferred by immobilization by staple fixation but not by denervation. Nonetheless, our findings suggest that bezafibrate may prevent muscle atrophy by suppressing expression of inflammatory cytokines associated with immobilization-induced muscle atrophy. Overall, we conclude that drug repositioning methods such as those used here are useful for screening drugs to prevent muscle atrophy, and that similar methods will be used to identify drugs in the future.

## Conclusions

Bezafibrate reduced immobilization-induced muscle atrophy by promoting decreases in macrophage-derived TNFα and phosphorylated Smad3 accumulation in muscle. These results suggest that bezafibrate may prevent muscle atrophy due to immobility and prevent further muscle atrophy.

### Supplementary Information

Supplementary Figures.

## Data Availability

The datasets used and/or analysed during the current study available from the corresponding author on reasonable request.
