# Supplementary material for: Bezafibrate attenuates immobilization-induced muscle atrophy in mice
Source: Sci Rep. 2024 Jan 26;14:2240. doi: 10.1038/s41598-024-52689-x (PMC10817916; doi:10.1038/s41598-024-52689-x)
Supplement: Supplementary file 1 — Supplementary Figures. [file 41598_2024_52689_MOESM1_ESM.pdf]

Bezafibrate attenuates immobilization-induced muscle atrophy in mice

Satoshi Nakamura[1], Yuiko Sato[1], Tami Kobayashi[1], Akihito Oya[1], Astuhiro Fujie[1], Morio Matsumoto[1], Masaya Nakamura[1], Arihiko Kanaji[1,*] and Takeshi Miyamoto[1,2,*]

[1]Department of Orthopedic Surgery, Keio University School of Medicine, 35 Shinano-machi, Shinjuku-ku, Tokyo 160-8582, Japan, [2]Department of Orthopedic Surgery, Kumamoto University, 1-1-1 Honjo, Chuo-ku, Kumamoto 860-8556, Japan

*Correspondence should be addressed to T.M. or A.K., Department of Orthopedic Surgery, Keio University School of Medicine, 35 Shinano-machi, Shinjuku-ku, Tokyo 160-8582, Japan

TEL: 81-3-5363-3812, FAX: 81-3-3353-6597, e-mail: Takeshi Miyamoto (miyamoto@z5.keio.jp or miyamoto.takeshi@kuh.kumamoto-u.ac.jp) or Arihiko Kanaji (hikokanaji@gmail.com)

Supplementary figure legends

Figure S1. Effects of 144 library compounds on inhibiting *Atrogin-1* expression in C2C12 myoblasts.

C2C12 cells were cultured for 24 hours in DMEM with (serum starvation, STRV-) or without (STRV+) 10% FBS in the presence or absence of indicated drugs (each 1 μM), and *Atrogin-1* expression was analyzed by realtime PCR. Data represent mean *Atrogin-1* expression relative to *Gapdh* ± SD. (n = 3 each).

Figure S2. Effects of bezafibrate treatment on Feret diameter in gastrocnemius and quadriceps muscles**.**

Nine-week-old C57BL/6 female mice were treated with bezafibrate or vehicle once daily for eight days, and staple fixation was performed on left hind limbs on day two. On day nine, mice were sacrificed and gastrocnemius and quadriceps muscles were harvested. Right hind limbs (sham-operated) served as controls (each group n = 5). We then assessed minimum Feret diameter of gastrocnemius (a) and quadriceps (c) muscle from bezafibrate-treated mice, with or without staple fixation, at three randomly selected regions. Data represent mean minimum Feret diameter of each muscle relative to vehicle-treated controls ± SD (each group n = 5. **P < 0.01, ***P < 0.001 by Student's t-test). Gastrocnemius (b) and quadriceps (d) muscle data are shown in boxplots (each group, n = 5. ***P < 0.001 by Student's t-test). The number of myofibers evaluated per mouse was 178-294 and 132-401 in gastrocnemius and quadriceps muscle, respectively, in the vehicle group, and in the bezafibrate group, 164-266 and 134-230 in gastrocnemius and quadriceps muscle, respectively.

Figure S3. Bezafibrate treatment decreases *Smad3* expression in stapled gastrocnemius.

(a-d) Nine-week-old C57BL/6 mice were treated with bezafibrate or vehicle once daily for eight days. Staple fixation was performed on left hind limbs on day two of drug treatment. On day nine, mice were sacrificed and gastrocnemius muscles were harvested, RNA was extracted, and realtime PCR analysis of relative levels of *Atrogin-1* (a), *MuRF1* (b), *Smad2* (c) and *Smad3* (d) transcripts was performed. Data represent mean gene expression relative to *Gapdh* ± SD (each n = 5, ***P < 0.001 by Student's t-test).

Figure S4. Biochemical testing of mouse blood.

Nine-week-old C57BL/6 female mice were administered bezafibrate or vehicle (ethanol) for eight days, and their sera were collected immediately before sacrifice. Creatine kinase (a), triglycerides (b), LDL- cholesterol (c), and HDL-cholesterol (d) levels were measured. Data represent means ± SD (each group n = 5. ns; not significant by Student's t-test).

Figure S5. Full-length western blots.

Full-length western blots of data shown in Fig. 3e. First antibodies were anti-pSmad2 and anti-pSmad3 (a), anti-Smad2/3 (b) and anti-Gapdh (c). Arrows show bands of indicated proteins.

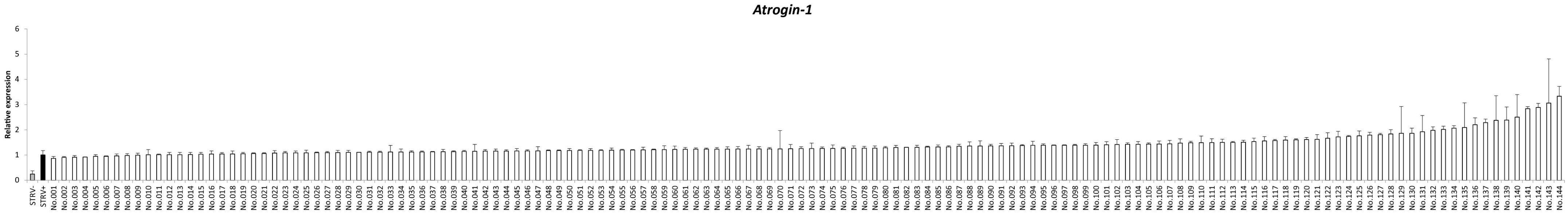

Supple1. Nakamura S. et al.

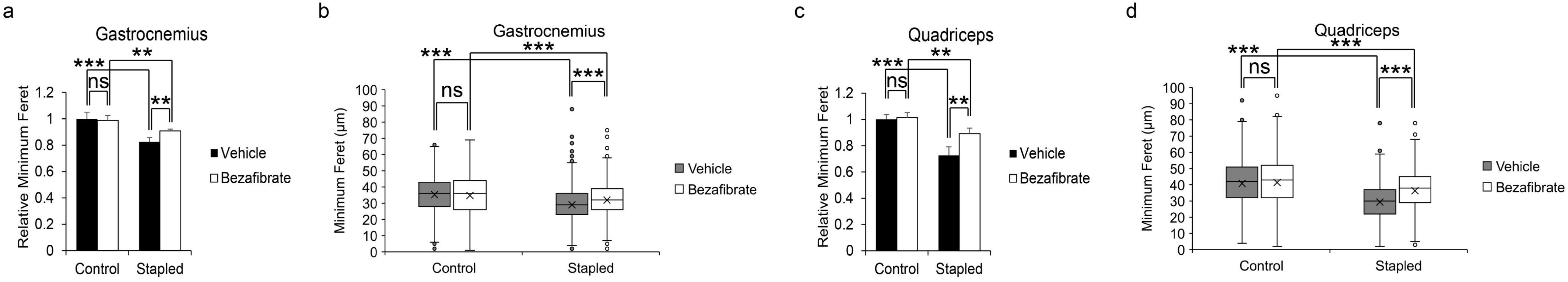

Supple2. Nakamura S. et al.

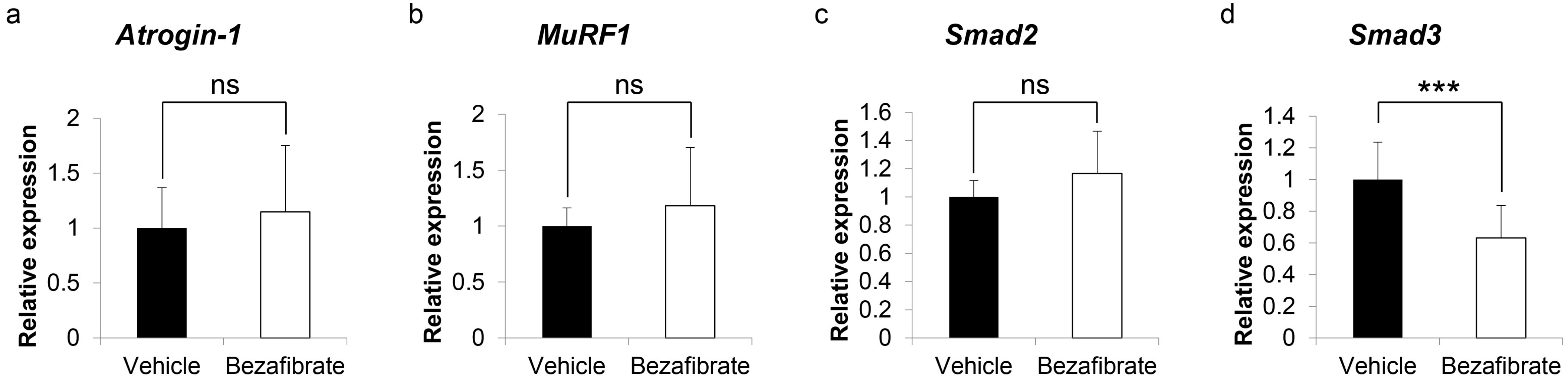

Supple3. Nakamura S. et al.

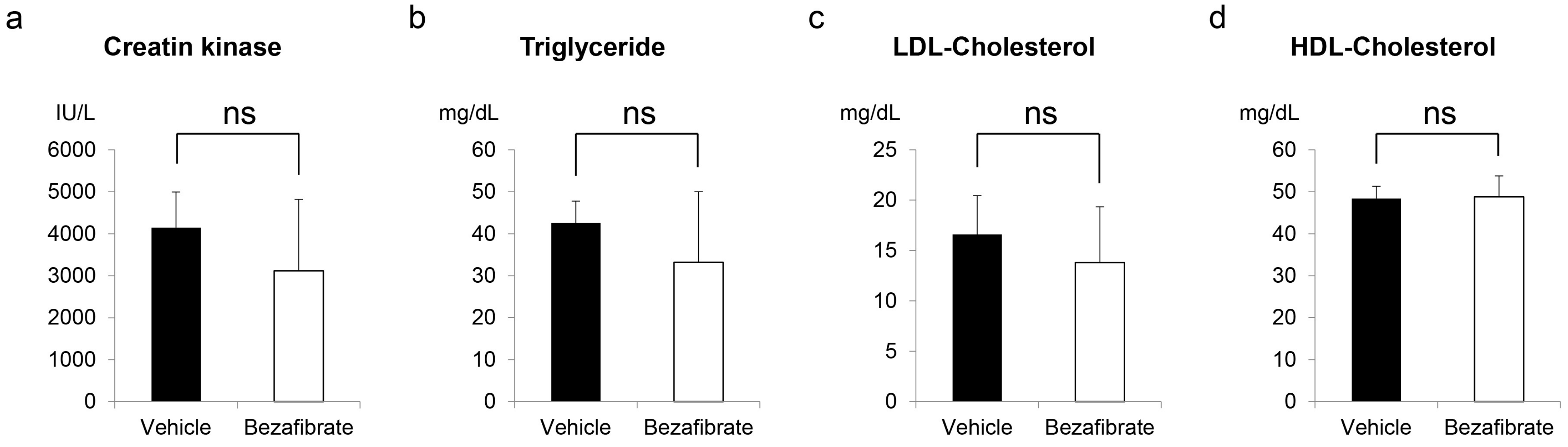

Supple4. Nakamura S. et al.

a pSmad2, pSmad3

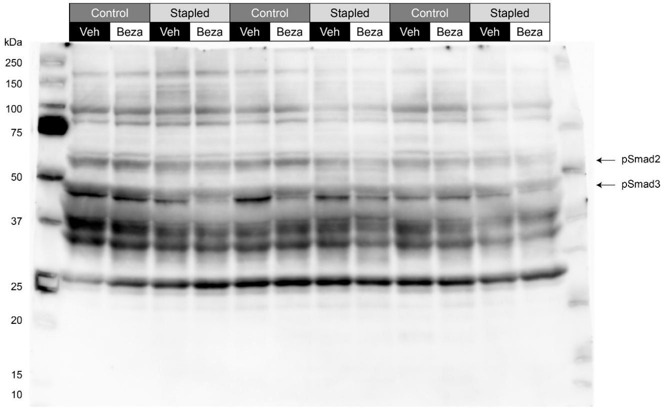

b Smad2, Smad3

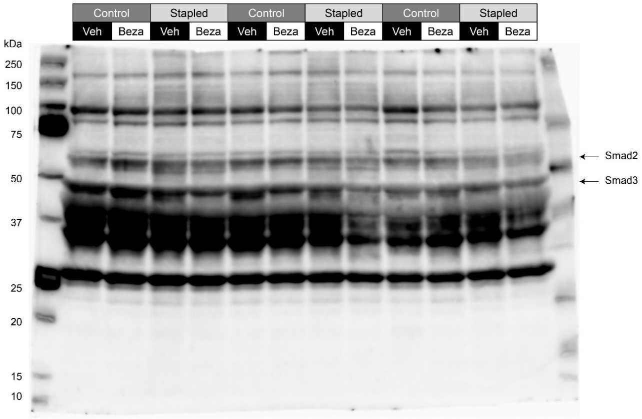

c Gapdh

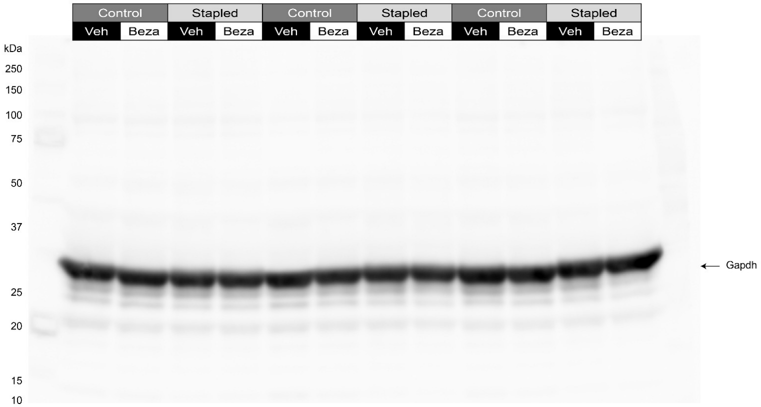

Supple2. Nakamura S. et al.
